# Effects of Adenovirus Type 5 E1A Isoforms on Viral Replication in Arrested Human Cells

**DOI:** 10.1371/journal.pone.0140124

**Published:** 2015-10-08

**Authors:** Sandi Radko, Richard Jung, Oladunni Olanubi, Peter Pelka

**Affiliations:** Department of Microbiology, University of Manitoba, 45 Chancellor’s Circle, Buller Building Room 427, Winnipeg, Manitoba, R3T 2N2, Canada; University of Regensburg, GERMANY

## Abstract

Human adenovirus has evolved to infect and replicate in terminally differentiated human epithelial cells, predominantly those within the airway, the gut, or the eye. To overcome the block to viral DNA replication present in these cells, the virus expresses the Early 1A proteins (E1A). These immediate early proteins drive cells into S-phase and induce expression of all other viral early genes. During infection, several E1A isoforms are expressed with proteins of 289, 243, 217, 171, and 55 residues being present for human adenovirus type 5. Here we examine the contribution that the two largest E1A isoforms make to the viral life cycle in growth-arrested normal human fibroblasts. Viruses that express E1A289R were found to replicate better than those that do not express this isoform. Importantly, induction of several viral genes was delayed in a virus expressing E1A243R, with several viral structural proteins undetectable by western blot. We also highlight the changes in E1A isoforms detected during the course of viral infection. Furthermore, we show that viral DNA replication occurs more efficiently, leading to higher number of viral genomes in cells infected with viruses that express E1A289R. Finally, induction of S-phase specific genes differs between viruses expressing different E1A isoforms, with those having E1A289R leading to, generally, earlier activation of these genes. Overall, we provide an overview of adenovirus replication using modern molecular biology approaches and further insights into the contribution that E1A isoforms make to the life cycle of human adenovirus in arrested human fibroblasts.

## Introduction

Human adenoviruses (HAdV) are a family of small, non-enveloped viruses with linear double-stranded DNA genomes of ~35 kilobase pairs. The viral genome is sub-divided into regions termed early and late, depending on when their transcription commences. Early genes are expressed in the initial stages of the infection, and late genes are expressed only after viral genome has begun to replicate [1]. The primary function of viral early genes is to remodel the intracellular environment in order to prepare the cell for viral reproduction (*i*.*e*. induction of S-phase), activation of viral gene expression, and evasion of the host immune response. The late genes constitute primarily viral structural proteins. The first gene expressed upon viral infection is the immediate early gene called Early Region 1A (*E1A*). The HAdV5 *E1A* gene encodes for two major proteins of 289 and 243 residues (R) that are expressed immediately after infection. These proteins arise from differential splicing of the same transcript and differ only by the presence of an internal sequence of 46 amino acids in the larger protein. At a later point in infection, splicing produces three additional mRNA species, which encode for proteins of 217, 171 and 55 amino acids [2–4]. Sequence comparisons of the largest E1A proteins of several adenovirus serotypes identified four regions of sequence conservation, designated conserved regions (CR) 1, 2, 3 and 4 [5–9].

HAdV5 E1A is a potent transcriptional regulator, yet it lacks the ability to directly bind to DNA. To overcome this limitation, E1A uses specific DNA-bound transcription factors to recruit itself to chromatin [10–13]. This results in alteration of chromatin structure via recruitment of transcriptional co-regulators leading to changes in gene expression [5, 14]. In the context of infection, the primary driver of viral gene expression is E1A289R, whereas E1A243R is generally considered a repressor [12, 15, 16]. Moreover, E1A289R and E1A243R are capable of affecting host gene expression [5], indeed E1A243R has been shown to modulate ~10,000 cellular promoters during infection [17, 18]. E1A243R causes a global redistribution of transcription factors, alteration of host gene expression, and genome-wide changes in epigenetic markers on chromatin [17, 19, 20]. Despite the fact that E1A289R is a potent transcriptional activator, little is known about how it changes cellular transcription.

The CR3 domain of HAdV5 E1A289R consists of residues 139–204, containing a C4 zinc finger domain that likely forms a defined structure and several sub-regions that are required for proper transcriptional activation and promoter targeting [5]. Paradoxically, studies of transcriptional reprogramming by E1A have largely focused on the predominantly transcriptionally repressive [12, 16] 243R isoform [17–19]. However, the primary driver of cellular reprogramming carried out by HAdV5 is E1A289R [21]. Our work on the properties of E1A has shown that there is a large gap in our understanding of how the different isoforms of E1A function [5, 13, 22]. The lack of understanding of how E1A289R is able to reprogram the infected cell leaves a significant void in our comprehension of E1A as an oncogene, particularly regarding its ability to induce cellular transformation and de-differentiation. We have previously identified a novel mechanism by which E1A289R is able to tether itself to cellular promoters via the use of DNA-bound transcriptional repressors [12, 13]. Specifically, E1A289R, but not 243R, stabilizes the repressive factor(s) on chromatin and overrides transcriptional silencing by recruitment of transcriptional co-activators, inducing gene expression.

In the present study we re-evaluate the contributions of different E1A isoforms to viral infection in arrested human cells using modern molecular biology approaches. In order to better understand how the major E1A isoforms contribute to viral growth in arrested human cells, we set out to determine how viruses that express E1A243R (*dl*520) or E1A289R (*pm*975) compare to a virus expressing all E1A isoforms (*dl*309) in viral growth, gene expression, and viral DNA replication. Here we show that viruses expressing E1A289R (*dl*309 and *pm*975) grow considerably better and faster than a virus lacking this isoform (*dl*520). Indeed, *dl*520 was never able to grow to the same degree as *dl*309 or *pm*975, suggesting an incomplete capacity by E1A243R to support virus replication. Viruses expressing E1A289R are able to drive viral gene and protein expression more efficiently, and are able to replicate their genomes faster and to greater numbers. Surprisingly, despite very different kinetics of infection and gene expression, induction of viral DNA replication occurred at similar times regardless of the E1A isoform present. We have also observed that the induction of cell cycle regulated genes occurs earlier when E1A289R is present. Our study provides further insights into how the different isoforms of E1A contribute to the viral life cycle and provides a modern view of the early events in viral infection under conditions that mimic the natural target of the virus, which are growth arrested and terminally differentiated cells.

## Materials and Methods

### Antibodies

Mouse monoclonal anti-E1A M73 and M58 antibodies were previously described [23] and were grown in-house and used as the hybridoma supernatant. Mouse monoclonal anti-72k DBP antibody was previously described [24] and was used at a dilution of 1:400 for western blot. Anti-Adenovirus type 5 (ab6982) antibody and anti-actin (ab3280) was purchased from Abcam and were used at the recommended dilutions. Secondary goat anti-mouse and goat anti-rabbit HRP-conjugated antibodies were purchased from Jackson Immunoresearch and were used at a dilution of 1:200,000. Note that the use of 4–12% Novex BOLT gels collapses post-translationally modified E1A species largely into bands corresponding to their respective sizes of 171, 243, and 289 residues, which we have consistently observed with both infection and transfection assays [12, 13, 16, 22].

### Cell and virus culture

IMR-90 cells (ATCC# CCL-186) were grown in Dulbecco’s Modified Eagle’s Medium (HyClone) supplemented with 10% fetal bovine serum (Invitrogen), streptomycin and penicillin (HyClone). To arrest IMR-90 cells, cells were grown to 100% confluence, and then incubated for an additional 72 hours to allow for complete growth arrest. All virus infections were carried out in serum-free media for 1 hour. Prior to infection, conditioned media from arrested IMR-90 cells was saved and was replaced back onto the cells after 1 hour incubation with the virus. All viruses used were grown in-house and were used at a multiplicity of infection (m.o.i.) of 5 for *dl*309, and 10 for *dl*520 and *pm*975. MG-132 (Sigma) was applied to cells at a final concentration of 10μM 4 hours prior to the indicated harvest time without changing the media.

### EdU incorporation assay

IMR-90 cells were grown until 100% confluent on LabTek II 4-chamber slides (Thermo-Fisher). After becoming fully confluent, cells were incubated for a further 72 hours to achieve growth arrest. Infections were carried out as described above with m.o.i. of 5 for *dl*309, and m.o.i. of 10 for *dl*520 and *pm*975. One hour prior to fixation, cells were pulsed with EdU for 1 hour as per manufacturer’s specifications using the Click-It EdU labeling kit for microscopy (Life Technologies). After EdU labeling, cells were fixed in 3.7% formaldehyde, stained for EdU using the Click-It kit with AlexaFluor 488, and labelled for E1A using M73 monoclonal antibody and AlexaFluor-594 conjugated secondary antibody (Jackson Immunoresearch). Cells were visualized using LSM700 laser confocal microscope and ZEN software suite. Quantification was carried out using ImageJ software.

### Real-time gene expression analysis

IMR-90 cells were infected with *dl*309, *dl*520, or *pm*975 at a variable m.o.i. to ensure equal E1A expression and at 8, 16, 24, and 72 hours after infections total cellular RNA was extracted using the TRIzol Reagent (Sigma) according to manufacturer’s instructions. 1.25 micrograms of total RNA was used in reverse-transcriptase reaction using SuperScript VILO reverse transcriptase (Invitrogen) according to the manufacturer’s guidelines using random hexanucleotides for priming. The cDNA was subsequently used for real-time expression analysis using the BioRad CFX96 real-time thermocycler. Fold changes in expression were determined by comparing expression levels of viral genes with *dl*309-infected cells at 24 hours after infection, while levels of cellular genes were compared to mock-infected cells. Analysis of expression data was carried out using the Pfaffl method [25] and was normalized to GAPDH levels. Primers used for *E1B*, *E2*, *E3*, *E4*, and *hexon* were previously described [26], and for *BLM*, *MCM4*, and *PCNA* in [13]. For detection of specific E1A splice isoforms the following primers were used: For 13S and 12S the forward primer was common binding in CR1: TTTTGAACCACCTACCCTTC, while the reverse primers were: 13S –CCACAGGTCCTCATATAGCAAA, and 12S –GGAGTCACAGCTATCCGTACTACT; for 11S and 10S the forward primer was common and the sequence is: GATCGAAGAGCCCGAGCA, while the reverse primers were the same as the reverse primers used for 13S and 12S, respectively. 9S splice product was detected with primers: TGATCGAAGAGGTCCTGTGTCT and TCAGGATAGCAGGCGCCA. Total E1A was detected with primers binding within exon 2, which is common to all E1A splice variants: TCCGGTCCTTCTAACACACC and GGCGTTTACAGCTCAAGTCC.

### Viral genome quantification

IMR-90 cells were lysed in lysis buffer (50mM Tris pH 8.1, 10mM EDTA and 1% SDS) on ice for 10 minutes. Lysates were sonicated briefly in a Covaris M220 focused ultrasonicator to break-up cellular chromatin and subjected to digestion using Proteinase K (NEB) according to manufacturer’s specifications. Following digestion viral DNA was purified using GeneJET PCR Purification Kit (Thermo-Fisher). PCR reactions were carried out using SYBR Select Master Mix for CFX (Applied Biosystems) according to manufacturer’s directions using 2% of total purified DNA as template according to manufacturer’s instructions using a CFX96 Real Time PCR instrument (BioRad). Standard curve for absolute quantification was generated by serially diluting pXC1 plasmid containing the left end of HAdV5 genome starting with a concentration of 1.0x10^7^ copies per reaction down to 1.0 copy per reaction. The primers used were the same as those used for expression analysis of *E1B* region, the annealing temperature used was 60°C and 40 cycles were run.

### Virus growth assay

Arrested IMR-90 cells were infected with HAdV5 *dl*309 [27], *pm*975 [21], or *dl*520 [28] viruses, at a variable m.o.i. specified above. Virus was adsorbed for one hour at 37°C under 5% CO_2_, after which cells were bathed in conditioned media and were re-incubated at 37°C under 5% CO_2_. Virus titres were determined 24, 48, 72, 96 and 120 h after infection, and plaque assays were performed on 293 cells by serial dilution. Prior to harvest, images of cells were acquired using phase-contrast optics on a Fisher Micromaster microscope.

## Results

### E1A isoforms affect virus replication

**Fig 1:**
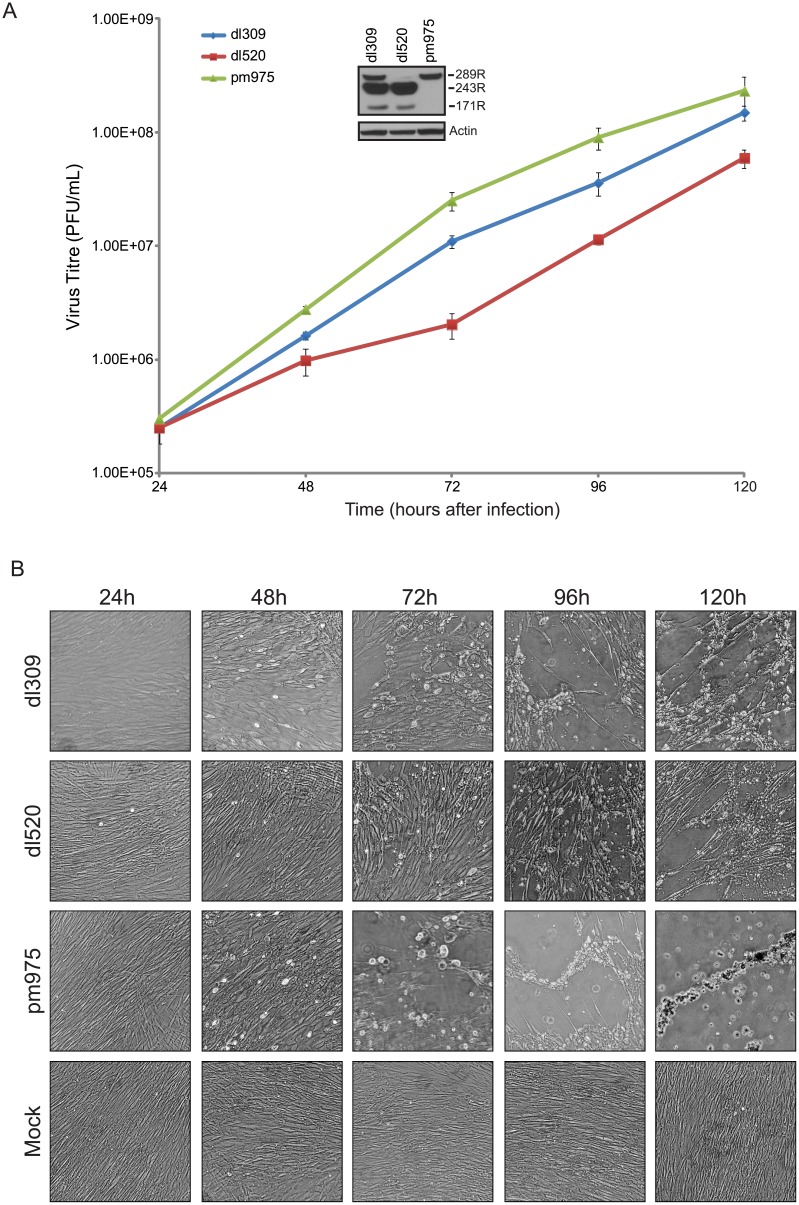
Virus growth and effects on cell morphology in arrested lung fibroblasts IMR-90. (A) IMR-90 cells were arrested by contact inhibition for three days. After which cells were infected with HAdV5 *dl*309, *dl*520, or *pm*975 for 1 hour in serum-free media. Media that was removed from the cells was saved and replaced after 1 hour. Virus titres were determined on 293 cells at the indicated time points. Inset shows E1A levels at 24 hours after infection. Error bars represent standard deviation (SD) of 4 replicate experiments. (B) Representative images of infected cells from A and mock-infected cells that were treated the same as infected cells, minus addition of virus. Images were taken prior to harvest of cells for titre determination and were taken at 100X magnification using phase-contrast optics.

Our previous work has shown that HAdV5 expressing different E1A isoforms (289R, 243R, or all E1As) have different abilities to drive arrested IMR-90 cells into S-phase [13]. Interestingly, viruses that express E1A289R were able to drive cells into S-phase much more quickly than viruses expressing only E1A243R. To determine whether these observed differences in S-phase induction translated into growth differences, we undertook virus growth analysis in arrested IMR-90 cells (Fig 1A). Since we were interested in the contribution of E1A to virus growth, we used infection conditions during which E1A protein levels are equal between viruses expressing different E1A isoforms. To achieve equal levels of E1A, cells were infected at m.o.i. of 5 for *dl*309, and m.o.i. of 10 for *dl*520 and *pm*975. To ensure that equal levels of E1A proteins were expressed as compared to *dl*309, we determined E1A levels at 24 hours after infection by western blot using the M73 antibody and quantifying the respective E1A bands (inset Fig 1A). IMR-90 cells were arrested by contact inhibition for 72 hours and infected with *dl*309 (expressing all E1A isoforms), *dl*520 (expressing E1A243R and E1A171R), or *pm*975 (expressing E1A289R and E1A217R). Viral titres were determined by plaque assays every 24 hours after initial infection starting at 24 hours and ending at 120 hours (Fig 1). Although all viruses replicated in arrested IMR-90 cells, those expressing E1A289R replicated quicker and to significantly higher titres. Interestingly *pm*975, which expresses only E1A289R (and E1A217R), replicated to the highest level. HAdV5 *dl*520, which does not express any E1A isoforms that contain CR3, showed consistently lower titres than either *pm*975 or *dl*309.

To assess the phenotypic effects of viral infection on arrested IMR-90 cells we monitored the cytopathic effect (CPE) and cellular morphology during the infection (Fig 1B). Uninfected cells showed unaltered morphology during the course of the experiment. Cells infected with *dl*309 or *pm*975 showed phenotypic changes associated with CPE much sooner than *dl*520 infected cells, at approximately 72 hours after infection. CPE was not observed in *dl*520 cells until approximately 96 hours after infection. Complete CPE was observed only in *pm*975-infected cells at 120 hours, while *dl*309-infected cells showed almost complete CPE at this time point. Cells infected with *dl*520 displayed considerably delayed CPE and at 120 hours resembled those observed at 96 hours for *dl*309- or *pm*975-infected cells. These observations show that the different E1A isoforms contribute differentially to virus growth, leading to significant differences in viral titres. In particular, viruses that express E1A289R were found to replicate significantly faster as compared to *dl*520, which does not express E1A289R.

### Viral genes are expressed differentially between viruses expressing different E1A isoforms

**Fig 2:**
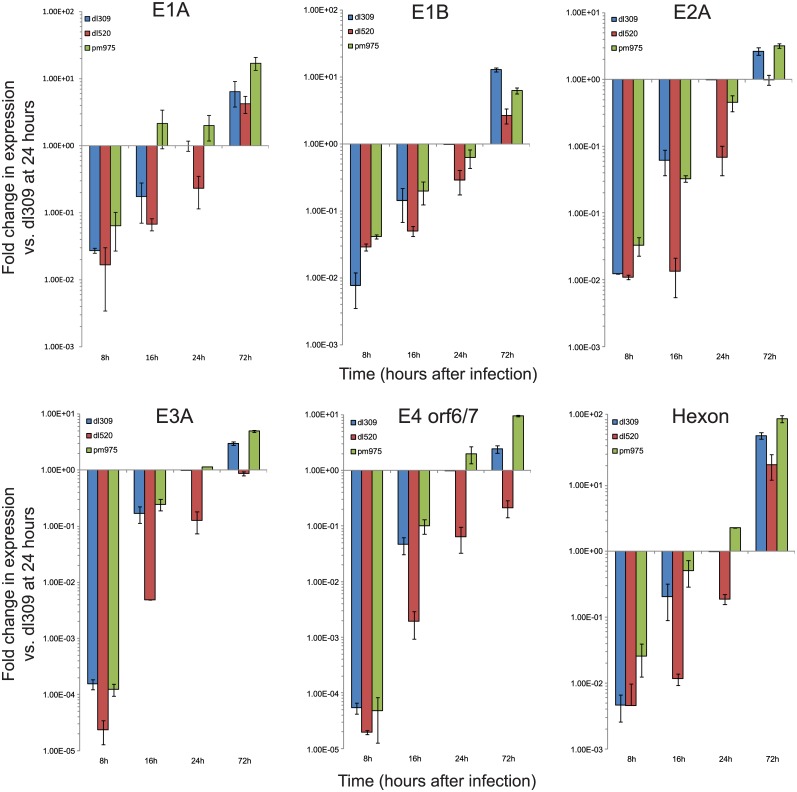
Expression of viral genes in arrested IMR-90 cells. IMR-90 cells were arrested by contact inhibition for three days. After which cells were infected with HAdV5 *dl*309, *dl*520, or *pm*975 for 1 hour in serum-free media. Media that was removed from the cells was saved and replaced after 1 hour. At the indicated time-points, total RNA was extracted using the TRIzol reagent and mRNA levels for the indicated genes were determined by qRT-PCR. GAPDH was used as a loading reference and viral gene expression was plotted with the levels detected for *dl*309-infected cells at 24 hours as a reference that was set to 1. Error bars represent SD of 4 biological replicates.

**Fig 3:**
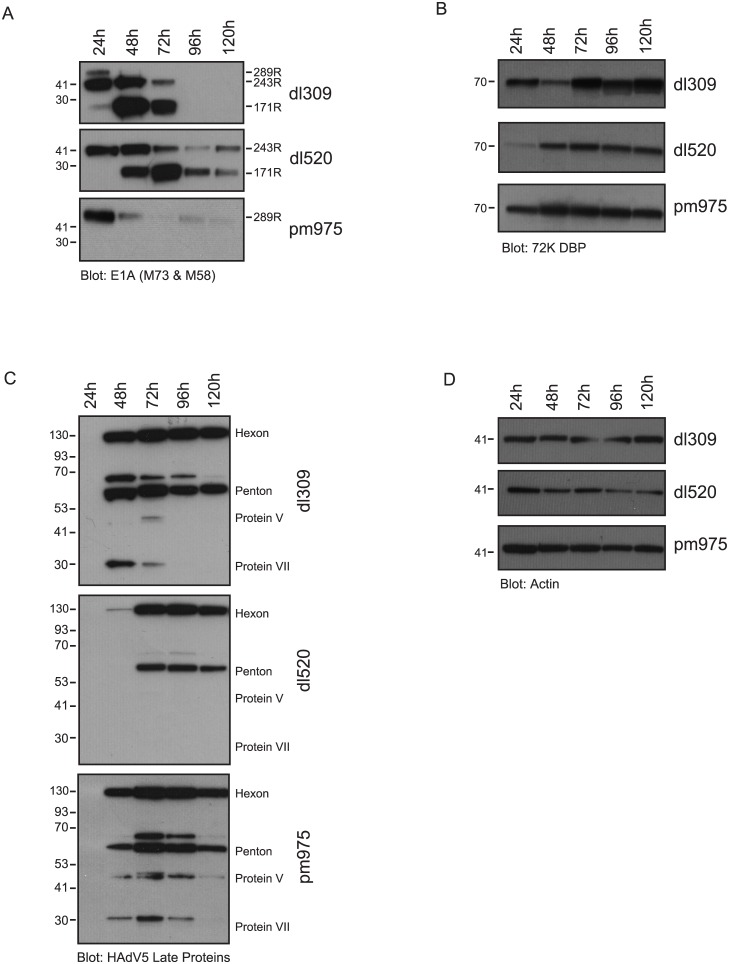
Viral protein levels after infection of arrested IMR-90 cells. (A) IMR-90 cells were arrested by contact inhibition for three days. After which cells were infected with HAdV5 *dl*309, *dl*520, or *dl*975 for 1 hour in serum-free media. Media that was removed from the cells was saved and replaced after 1 hour. At the indicated time-points, cells were lysed and 20μg total cellular lysate was resolved by SDS-PAGE on Novex BOLT 4–12% gradient minigel. E1A was detected with a combination of M58 and M73 monoclonal antibodies and visualized using a secondary HRP-conjugated anti-mouse antibody (Jackson Immunoresearch). (B) Same as A except probed for the 72kDa E2 DNA-binding protein. Secondary HRP-conjugated anti-mouse antibody (Jackson Immunoresearch) was used for detection. (C) Same as A except probed with antibody recognizing viral structural proteins (Abcam). Secondary anti-rabbit HRP-conjugated antibody was used for detection (Jackson Immunoresearch). (D) Same as A except probed for cellular actin (Abcam) as a loading control. Secondary anti-rabbit HRP-conjugated antibody was used for detection (Jackson Immunoresearch).

To further determine how the different E1A isoforms contribute to viral replication, we set out to determine how viral early and late genes were expressed, and the levels of early and late viral proteins during infection of arrested IMR-90 cells. RNA samples from arrested IMR-90 cells were collected at 8, 16, 24, and 72 hours after infection with *dl*309, *dl*520, or *pm*975 (Fig 2). Because the cells do not express any viral genes prior to infection, we compared the different infections and time points to *dl*309-infected IMR-90 cells at 24 hours after infection (Fig 3). This time point was selected because all viral transcripts were readily detectable by qRT-PCR.

E1A transcripts were readily detectable at 8 hours after infection in *dl*309 and *pm*975 infected cells, but were only slightly above background in *dl*520 infected cells. E1A mRNA levels continued to increase steadily for all viruses, however at different rates. *dl*520-infected cells expressed the lowest levels of E1A until 72 hours after infection when they reached levels comparable to those observed in *dl*309 infected cells. The levels of other viral mRNAs, including E1B, E2A, E3A, E4 orf6/7, and hexon, paralleled those observed for E1A, with *dl*309 and *pm*975 having generally higher levels than *dl*520-infected cells (Fig 2). These observations also correlate well with the observed overall growth results, with *dl*309 and *pm*975 replicating similarly, while *dl*520 replicated at a slower rate.

To determine whether viral mRNA levels correlated with viral protein levels, we also performed western blot analysis of infected cells at 24, 48, 72, 96, and 120 hours after infection (Fig 3). Overall, the mRNA levels were closely related to the level of viral proteins with a few exceptions. E1A mRNA levels continued to increase steadily throughout the course of the infection for all viruses (Fig 2), but E1A protein levels peaked early on in the infection, between 24 and 48 hours depending on the virus, followed by a steady decline. This was particularly apparent for the E1A289R isoform, in both *dl*309 and *pm*975-infected cells. In the case of *pm*975-infected cells, E1A was expressed at the highest level at 24 hours after infection, and was largely undetectable by 72 hours, while the levels of E1A mRNA continued to climb. Similarly, E1A289R was detectable only at 24 hours after infection of IMR-90 cells with *dl*309, and was not detectable later on in the infection. Interestingly, we have also observed a shift in E1A isoforms expressed, with E1A243R and E1A171R being the predominant isoforms 48 hours and later after infection with *dl*309.

Levels of other viral proteins examined (E2A DNA-binding protein (DBP) and viral structural proteins) correlated closely with their respective mRNAs (Fig 3). The levels of DBP and hexon were lower in *dl*520-infected cells as compared to those infected with *dl*309 or *pm*975 at a given time point. For example, DBP was only readily detectable in *dl*520-infected cells at 48 hours after infection, with only a faint band observed at 24 hours (Fig 3B). Similarly, structural proteins were not readily detectable in *dl*520-infected cells until 72 hours after infection. In fact, some late proteins (such as Proteins V and VII) were never detectable in *dl*520-infected cells by western blot. Together, these observations demonstrate significant differences in the rates of viral infection of arrested IMR-90 cells.

### E1A isoform levels during the course of infection

**Fig 4:**
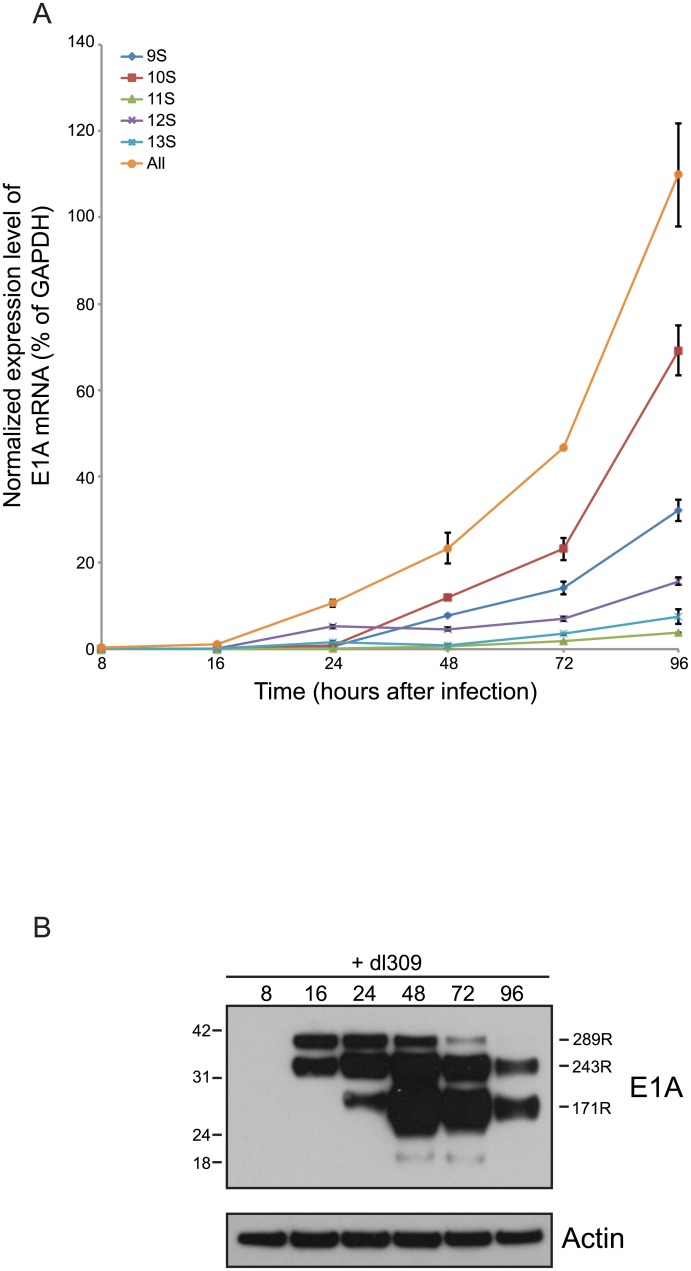
E1A isoform splicing and protein levels in *dl*309-infected IMR-90 cells. (A) Contact-inhibited IMR-90 cells were infected with *dl*309 at an m.o.i. of 5, at the indicated time-points total RNA was extracted from the cells using the TRIzol reagent and mRNA levels for specific E1A splice isoforms were determined by qRT-PCR using primers designed to recognized only the targeted E1A splice variant. GAPDH was used as a loading reference. E1A mRNA levels are represented as fraction of GAPDH levels at the given time-point. Error bars represent SD of 4 biological replicates. (B) Arrested IMR-90 cells were infected with *dl*309 as in (A). Four hours prior to the indicated time-point cells were treated with 10μM MG-132 for 4 hours. Total protein was extracted and resolved on gradient SDS-PAGE. E1A was detected using M58 and M73 monoclonal antibodies. Actin was used as a loading control. Secondary anti-mouse HRP-conjugated antibody was used for detection.

Since we have observed that E1A289R protein levels drop precipitously after 24 hours of infection (Fig 3A) we wanted to determine whether this is due to the reduction of the 13S mRNA, translational effects, or enhanced protein degradation. To do this, we analyzed levels of each E1A mRNA during the course of infection in *dl*309-infected arrested human fibroblasts (Fig 4) using real-time primers specific for each E1A mRNA. Overall E1A levels continued to climb throughout the infection (Fig 4A, orange line), while levels of 12S and 13S mRNA (Fig 4A, purple and light blue, respectively) peaked 24 hours after infection and remained relatively steady until the end of the assay. Interestingly, levels of 12S mRNA were approximately 3-fold higher than 13S mRNA (5.2% vs. 1.5% of GAPDH levels) while levels of other E1A transcripts were negligible at 24 hours after infection. 48 hours after infection, levels of 12S and 13S mRNA remained similar to those at 24 hours after infection and stayed at this approximate level until the end of the assay. Interestingly, at 48 hours we observed an increase in 10S and 9S transcripts (Fig 4A). Indeed, these two transcripts represented the bulk of E1A expression 48 hours and onwards after infection, with the 10S transcript being the most abundant E1A transcript present during infection. The 11S mRNA remained low throughout the infection, staying at a level equal to or lower than 13S mRNA.

The steady levels of 13S and 12S mRNAs during the infection suggest that the reduced protein level is due to either preferential translation of the smaller E1A mRNAs or due to specific degradation of the 289R and 243R E1A isoforms. To examine the latter possibility, we have used the proteasome inhibitor MG-132 [29] to see whether E1A289R and E1A243R levels can be restored (Fig 4B). MG-132 was applied to infected cells for 4 hours, 4 hours before the indicated time point and E1A levels were analyzed by western blot. MG-132 treatment restored the 289R protein up to 72 hours after infection and overall increased levels of the 243R protein, suggesting that degradation of the larger E1A isoforms was, at least, partly responsible for the reduced protein levels observed after 24 hours of infection. Interestingly, levels of 171R E1A were the highest and paralleled mRNA levels while E1A217R was not detected. Curiously, we have also observed a small, ~18kDa, species of E1A late in the infection (Fig 4B). It is unclear what this species is; since we have used the M58 and M73 antibodies for detection it cannot be the 55R product as this E1A protein lacks the epitopes for these two antibodies. However, it may represent a partial degradation product of larger E1A proteins.

Together, these results provide a clear picture of E1A mRNA levels during the course of viral infection of arrested human fibroblasts.

### Viral DNA replication in the infected cell

**Fig 5:**
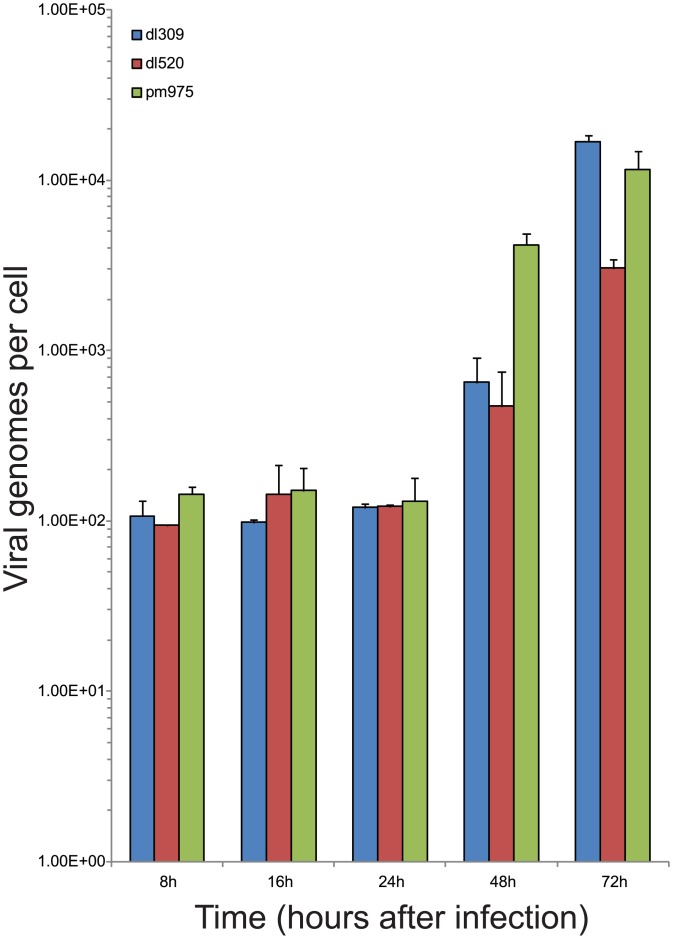
Viral genome levels in infected IMR-90 cells. IMR-90 cells were arrested by contact inhibition for three days. After which cells were infected with HAdV5 *dl*309, *dl*520, or *pm*975 for 1 hour in serum-free media. Media that was removed from the cells was saved and replaced after 1 hour. At the indicated time points cells were harvested and genomes were quantified on per cell basis using qPCR for the viral E1B gene. pXC1 plasmid was used for standard curve generation and absolute copy number quantification. Error bars represent SD of 4 biological replicates.

In order to determine the contribution that E1A isoforms make to the kinetics of viral DNA replication, we assessed viral genome copy number in infected, arrested IMR-90 cells. Viral genomes were quantified at 8, 16, 24, 48, and 72 hours after infection as previously described [26]. Viral DNA replication was not observed until some time between 24 and 48 hours after infection (Fig 5). Surprisingly, all viruses entered this phase of the viral life cycle at similar time-points, although the number of viral genome copies differed, reflecting, perhaps, the efficiency of viral genome replication. This is considerably later than what has been previously observed in transformed cells [26, 30], but is consistent with reports in normal cells [31]. The number of genomes observed was steady at approximately 100 copies/cell up to 24 hours after infection, likely reflecting the initial virus input and the limitations of the assay to resolve small differences in initial genome load (Fig 5). At 48 hours after infection, there was a significant increase in genomes per cell, with *dl*309 and *dl*520 having approximately 500 genomes/cell, and *pm*975 having approximately 5,000 genomes/cell. Genome copy number increased at 72 hours to over 10,000 genomes/cell for *dl*309 and *pm*975, and about 3,000 for *dl*520. Collectively, these results show that all viruses enter the late phase of their replicative cycle between 24 and 48 hours after infection, but they differ significantly in the efficiency of viral DNA replication.

### Effects of E1A isoforms on cell cycle regulated gene expression and S-phase induction

**Fig 6:**
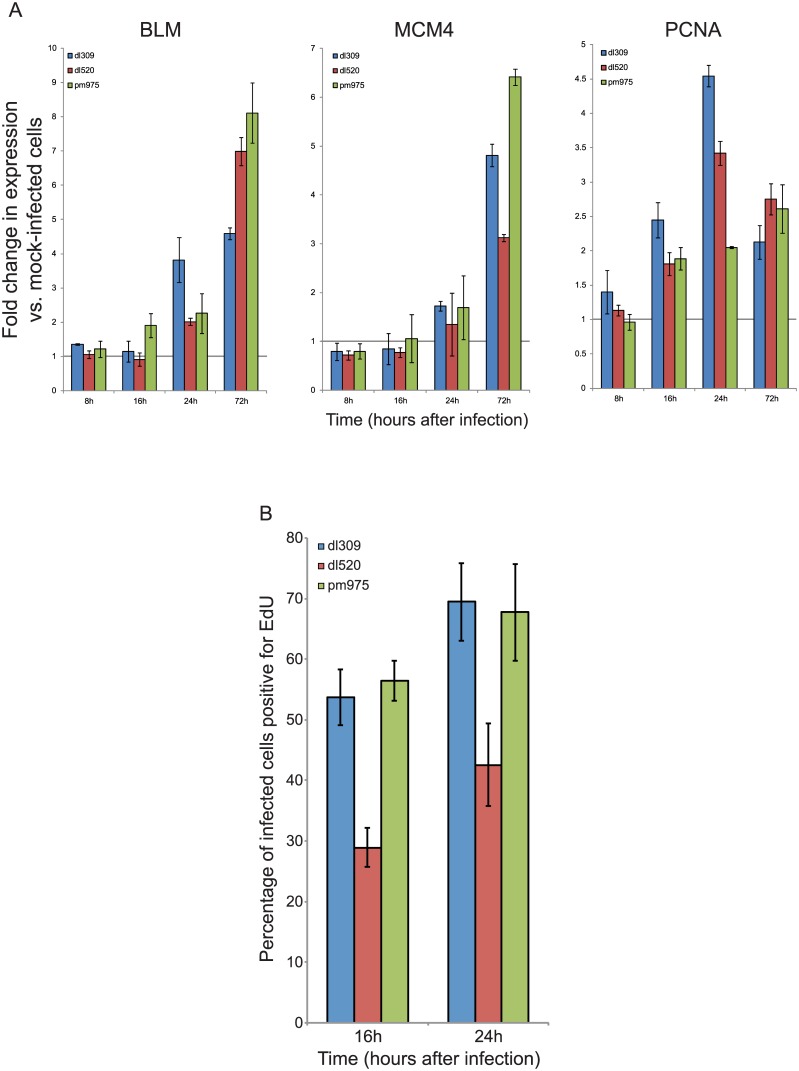
Expression of cellular S-phase specific genes in infected IMR-90 cells. (A) IMR-90 cells were arrested by contact inhibition for three days. After which cells were infected with HAdV5 *dl*309, *dl*520, or *pm*975 for 1 hour in serum-free media. Media that was removed from the cells was saved and replaced after 1 hour. At the indicated time-points, total RNA was extracted using the TRIzol reagent and mRNA levels for the indicated genes were determined by qRT-PCR. GAPDH was used as a loading reference and mock-infected cells were used as a control and were set to 1. Error bars represent SD of 4 biological replicates. (B) IMR-90 cells were arrested by contact inhibition for three days. After which cells were infected with HAdV5 *dl*309, *dl*520, or *pm*975 for 1 hour in serum-free media. Media that was removed from the cells was saved and replaced after 1 hour. One hour prior to the indicated time point cells were pulsed with EdU for 1 hour, fixed at the indicated time point, and stained for EdU and E1A as described in the Materials and Methods. Data represents percentage of infected cells that were positive for EdU staining. Error bars represent SD of 5 biological replicates.

Our previous work has shown that E1A289R alone is as efficient at driving arrested human fibroblasts into S-phase as viruses expressing all E1A isoforms, whereas cells infected with viruses expressing only E1A243R were not as capable [13]. Previously, we have also observed that induction of cell-cycle specific genes was the highest in *pm*975-infected cells, and the lowest in *dl*520-infected cells at 24 hours after infection. In order to obtain a clearer picture of deregulation of cell-cycle specific genes in infected cells, we analysed expression of three genes regulated by E2Fs [13, 32, 33] during the course of infection (Fig 6A). Expression of *PCNA* is not only E2F-regulated but has previously been shown to be upregulated by E1A [34–36]. Expression of *BLM*, *MCM4*, and *PCNA* was unaltered at 8 hours after infection as compared to mock-infected cells. At 16 hours after infection, BLM transcript levels increased slightly in *pm*975-infected cells, but not others, whereas PCNA mRNA was elevated for all infections. At 24 hours after infection, levels of all three transcripts were elevated in *dl*309 infected cells, and they continued to increase with the exception of PCNA mRNA, which was reduced at 72 hours after infection as compared to 24 hours. Interestingly, levels of PCNA mRNA were induced the least by *pm*975 infection at 24 hours. Overall, infection with *dl*309 induced expression of *BLM*, *MCM4*, and *PCNA* to levels higher than *dl*520 infection, and with the exception of *PCNA* to levels similar to those observed in *pm*975-infected cells. Analysis of S-phase induction by EdU incorporation assay (Fig 6B) showed differences in the ability of the specific viruses to drive cellular DNA replication. In particular, we observed that *dl*520, which lacks E1A289R, was deficient in S-phase induction as compared to *dl*309 or *pm*975, particularly at 16 hours after infection. In conclusion, all viruses were able to activate transcription of these cell cycle regulated genes, which was consistent with their ability to induce S-phase.

## Discussion

Human adenovirus expresses five different E1A mRNA species during the course of viral infection, with proteins of 289R, 243R, 217R, 171R, and 55R being present at some point during infection with HAdV5 [5]. Two of the primary functions of E1A during infection are activation of viral early gene expression and induction of cellular S-phase, which enable the viral genome to be efficiently replicated. The contribution of E1A289R to viral infection has not been thoroughly examined. Previous work [21, 30, 37, 38] comparing growth of viruses expressing all E1A isoforms versus those expressing only E1A289R or E1A243R showed some differences in how these viruses behave, however a comprehensive study on viral growth, gene expression, protein levels, and viral DNA replication in arrested human cells has not been carried out. Here we report an analysis of the kinetics of how viruses expressing all E1A isoforms (*dl*309), E1A289R (*pm*975), or E1A243R (*dl*520) replicate, express their genes and proteins, copy their DNA, and drive expression of select cell-cycle regulated genes in arrested lung fibroblasts.

All viruses replicated in arrested IMR-90 cells (Fig 1); however, *dl*520, lacking E1A289R or E1A217R, replicated slower than *dl*309 and *pm*975. On average, this virus trailed by about 24 hours in terms of viral titres (Fig 1), and viral gene (Fig 2) and protein expression (Fig 3). Furthermore, given sufficient time to drive cells to complete CPE, *dl*520 never achieved titers as high as those observed with *dl*309 or *pm*975 (data not shown), suggesting a fundamental inability of E1A243R to fully support viral replication. Surprisingly, *pm*975, expressing only E1A289R, replicated slightly faster than *dl*309 virus that expresses all E1A isoforms, with the last time-point showing convergence between *dl*309 and *pm*975. This result is somewhat different from previous reports [30]. Importantly, *pm*975 behaved similarly to *dl*309 in several metrics used to assess how the virus is performing, such as gene expression and induction of CPE (Figs 2, 3 and 6). Several factors may contribute to the observed differences in viral growth between the viruses studied. In earlier studies of viral replication kinetics, the goal was to compare the infection at identical m.o.i. across the different viruses, whereas we have used conditions that lead to the expression of similar E1A levels at 24 hours after infection (Figs 1 and 3A) in order to compare E1A effects more directly. Even under these conditions, the m.o.i. that we have used was similar for all viruses. Furthermore, the use of WI-38 cells in earlier studies may contribute to the observed differences. Although both of these cell lines are derived from the same cell type and tissue [39, 40], they originate from different donors and therefore have different genetic backgrounds. Indeed, our examination of viral protein levels in arrested WI-38 cells showed reduced protein levels compared to IMR-90 cells (data not shown), indicating that even under identical infection conditions there are clear differences in how these cells support viral replication. Lastly, the lack of the E1A289R in *dl*520-infected cells will lead to reduced viral gene transactivation, as we have observed here and was previously reported [37, 38].

Examination of viral protein levels has shown some interesting and critical differences between viruses that express all E1A isoforms, and those expressing either E1A289R (*pm*975), or E1A243R (*dl*520) (Fig 2). E1A levels were similar between the different infections at 24 hours after infection (as expected since we optimized infection conditions for this purpose), but varied significantly across time and virus type. Levels of E1A289R dropped early in the infection, and E1A243R saw reduction after viral genome replication commenced (Figs 3 and 5). Somewhat unexpectedly, we observed increasing levels of E1A171R (derived from the 10S mRNA) in *dl*309 and *dl*520 infected cells, whereas levels of E1A217R (derived from the 11S mRNA) were not detectable. Interestingly, the amount of E1A mRNA continued to climb throughout the course of infection (Figs 2 and 4), indicating that the differences in protein expression are likely a result of splicing site selection changes, translational effects, or isoform specific degradation. Since 12S and 13S mRNA levels remained relatively steady after 24 hours in infected cells and inhibition of the proteasome restored 243R and 289R E1A protein expression (Fig 4B), it is not splicing but likely translational rates and protein stability that dictate levels of 289R and 243R E1A in infected cells. It is curious to observe high levels of 10S mRNA and the 171R E1A protein in the infected cell since this protein has previously been shown to supress virus growth [41]. Considering that E1A171R is abundant later on in the infection, but is not expressed early on, it is probable that the suppressive effects occur early during infection, when predominantly E1A289R and E1A243R are present. Indeed, E1A171R has been shown to interfere with the ability of E1A to induce S-phase and transform cells [41].

Viral late and structural proteins were expressed at lower levels in *dl*520-infected cells as compared to those infected with *dl*309 or *pm*975 (Fig 3C). Unexpectedly, certain late proteins were not detectable by standard western blot in *dl*520-infected cell lysates (Fig 3C). For example, Protein VII was undetectable in *dl*520-infected cells, despite high levels of viral mRNAs and high viral genome copy number. This was somewhat unexpected as this protein is essential for virus growth [42], it is likely that sufficient quantities of the protein were made to support virus replication but not enough to be detectable by standard western blot. Nevertheless, these observations suggest that E1A289R is required for full activation of viral late gene transcription. This is not unexpected, as we have previously observed recruitment of E1A to the viral major late promoter during infection [26]. It should be noted that we did not observe a dramatic decrease in GAPDH mRNA levels throughout the infection, although we did observe a slight drop (approximately equal to one Ct) very late in the infection (96 hours and later for *dl*309 and *pm*975, even later for *dl*520). Since most of our real-time data was analyzed during earlier time points (except for Fig 4A), this has little bearing on our findings. In fact, inhibition of *GAPDH* expression could be deleterious to the virus as this is an enzyme essential for glycolysis, which can provide energy required for virus growth. Therefore, it is logical to assume that the virus would be somewhat selective in the types of host genes shut off even late in infection, as observed for other genes earlier in infection [43, 44].

Previous studies [13, 21] have shown that *pm*975 is as efficient at inducing cellular DNA replication as *dl*309 in arrested fibroblasts, contrary to other studies where *pm*975 was shown to be deficient [30]. Induction of cellular DNA synthesis is a pre-requisite for viral DNA replication, despite the virus producing its own DNA polymerase. Analysis of viral genome replication showed that all viruses are capable of replicating their DNA, starting between 24 and 48 hours after infection, which was consistent with earlier reports for HAdV5 and HAdV2 [30, 31]. Interestingly, there was little difference in the time at which the viral genome began to replicate between the different viruses (Fig 5). This observation suggests that cellular intrinsic factors govern viral DNA replication, rather than extrinsic or virus-specific factors, such as levels of the viral DNA replication machinery. This proposition is further supported by our observations of earlier DNA replication in HT1080 cells [26], where DNA replication commenced between 10 and 20 hours after infection at similar m.o.i. HT1080 cells are transformed and continually growing, therefore their supply of cellular co-factors required for DNA replication is constantly high and does not restrict viral genome replication. Consequently, viral DNA replication will be initiated as soon as the virus is ready rather than when the cell is. In arrested IMR-90 cells the levels of viral DNA replication machinery may be sufficient for copying of viral DNA, but cellular environment may not be permissive.

To correlate viral genome replication with cellular S-phase specific genes we have examined the level of expression of three cellular genes that are strongly induced at the onset of S-phase [13]; *BLM*, *MCM4*, and *PCNA* (Fig 6). Induction of cellular genes correlated with the induction of viral DNA synthesis (Figs 5 and 6). It is interesting to note that PCNA mRNA levels were elevated earlier (Fig 6), possibly due to its role in DNA damage response [45] that may be induced by unscheduled cellular DNA replication. We have also observed that PCNA mRNA levels decrease at 72 hours after infection, perhaps due to the same reasons as the observed early expression of *PCNA* during the infection. Although BLM has previously been implicated in double-strand DNA break repair [46], its mRNA levels did not decrease nor were they elevated early in infection. This may be due to the type of DNA damage response in which PCNA and BLM participate, where PCNA is largely involved in response to stalled replication forks (reviewed in [45]), while BLM participates in double-strand repair by unwinding DNA and recruitment of DNA2 [47]. It is possible that the former response is deleterious to viral DNA replication, while the latter is either beneficial or has no effect. Nevertheless, our results highlight the differences in how E1A isoforms contribute to the induction of cell-cycle specific genes during infection, showing that E1A289R is required for maximal expression. The higher induction of E2F-regulated genes by E1A289R may also be due to the more efficient induction of the viral E4 promoter driving expression of *E4 orf6/7* (Fig 2), which can independently induce E2F-regulated genes [48, 49].

Recently, E1A289R has not been the primary focus of E1A research. However, our previous work [12, 13, 16, 22] has clearly shown the importance of this isoform in the viral life cycle and induction of S-phase. We have examined how viruses expressing different E1A isoforms replicate in arrested human lung fibroblasts, highlighting critical differences. Specifically, viruses expressing E1A289R were considerably more efficient in carrying out the viral life cycle than *dl*520, lacking this isoform entirely. Importantly, replication of *dl*520 never reaches the same levels of viral yield as those observed for *dl*309 or *pm*975, suggesting a fundamental defect in the viral life cycle since initial virus input should not affect final virus output under ideal conditions (such as those seen in cell culture models as used here). Much remains to be discovered about the contribution that E1A289R makes to deregulation of cellular growth, but our studies have so far highlighted the differences in how this E1A isoform behaves compared to the others, providing greater understanding of deregulation of cellular growth by adenovirus.

## References

[1] Knipe DM, Howley PM. Fields virology. 6th ed Philadelphia, PA: Wolters Kluwer/Lippincott Williams & Wilkins Health; 2013 2 volumes p.

[2] Miller MS, Pelka P, Fonseca GJ, Cohen MJ, Kelly JN, Barr SD, et al Characterization of the 55-residue protein encoded by the 9S E1A mRNA of species C adenovirus. J Virol. 2012;86(8):4222–33. Epub 2012/02/04. 10.1128/JVI.06399-11 22301148 PMC3318620

[3] Ulfendahl PJ, Linder S, Kreivi JP, Nordqvist K, Sevensson C, Hultberg H, et al A novel adenovirus-2 E1A mRNA encoding a protein with transcription activation properties. EMBO J. 1987;6(7):2037–44. Epub 1987/07/01. 2958277 10.1002/j.1460-2075.1987.tb02468.x PMC553593

[4] Stephens C, Harlow E. Differential splicing yields novel adenovirus 5 E1A mRNAs that encode 30 kd and 35 kd proteins. EMBO J. 1987;6(7):2027–35. Epub 1987/07/01. 2958276 10.1002/j.1460-2075.1987.tb02467.x PMC553592

[5] Pelka P, Ablack JN, Fonseca GJ, Yousef AF, Mymryk JS. Intrinsic structural disorder in adenovirus E1A: a viral molecular hub linking multiple diverse processes. J Virol. 2008;82(15):7252–63. Epub 2008/04/04. JVI.00104-08 [pii] 10.1128/JVI.00104-08 . 18385237 PMC2493305

[6] van Ormondt H, Maat J, van Beveren CP. The nucleotide sequence of the transforming early region E1 of adenovirus type 5 DNA. Gene. 1980;11(3–4):299–309. . 6260576 10.1016/0378-1119(80)90070-0

[7] Avvakumov N, Wheeler R, D'Halluin JC, Mymryk JS. Comparative sequence analysis of the largest E1A proteins of human and simian adenoviruses. J Virol. 2002;76(16):7968–75. Epub 2002/07/23. 12134001 10.1128/JVI.76.16.7968-7975.2002 PMC155151

[8] Avvakumov N, Kajon AE, Hoeben RC, Mymryk JS. Comprehensive sequence analysis of the E1A proteins of human and simian adenoviruses. Virology. 2004;329(2):477–92. . 15518825 10.1016/j.virol.2004.08.007

[9] Kimelman D, Miller JS, Porter D, Roberts BE. E1a regions of the human adenoviruses and of the highly oncogenic simian adenovirus 7 are closely related. J Virol. 1985;53(2):399–409. . 3968719 10.1128/jvi.53.2.399-409.1985 PMC254650

[10] Liu F, Green MR. A specific member of the ATF transcription factor family can mediate transcription activation by the adenovirus E1a protein. Cell. 1990;61(7):1217–24. . 2142019 10.1016/0092-8674(90)90686-9

[11] Liu F, Green MR. Promoter targeting by adenovirus E1a through interaction with different cellular DNA-binding domains. Nature. 1994;368(6471):520–5. Epub 1994/04/07. 10.1038/368520a0 . 8139685

[12] Bruton RK, Pelka P, Mapp KL, Fonseca GJ, Torchia J, Turnell AS, et al Identification of a second CtBP binding site in adenovirus type 5 E1A conserved region 3. J Virol. 2008;82(17):8476–86. Epub 2008/06/06. JVI.00248-08 [pii] 10.1128/JVI.00248-08 . 18524818 PMC2519622

[13] Pelka P, Miller MS, Cecchini M, Yousef AF, Bowdish DM, Dick F, et al Adenovirus E1A directly targets the E2F/DP-1 complex. J Virol. 2011;85(17):8841–51. Epub 2011/07/01. JVI.00539-11 [pii] 10.1128/JVI.00539-11 21715488 PMC3165805

[14] Berk AJ. Recent lessons in gene expression, cell cycle control, and cell biology from adenovirus. Oncogene. 2005;24(52):7673–85. Epub 2005/11/22. 1209040 [pii] 10.1038/sj.onc.1209040 . 16299528

[15] Dery CV, Herrmann CH, Mathews MB. Response of individual adenovirus promoters to the products of the E1A gene. Oncogene. 1987;2(1):15–23. . 2963989

[16] Pelka P, Ablack JN, Torchia J, Turnell AS, Grand RJ, Mymryk JS. Transcriptional control by adenovirus E1A conserved region 3 via p300/CBP. Nucleic Acids Res. 2009;37(4):1095–106. Epub 2009/01/09. gkn1057 [pii] 10.1093/nar/gkn1057 . 19129215 PMC2651774

[17] Ferrari R, Gou D, Jawdekar G, Johnson SA, Nava M, Su T, et al Adenovirus small E1A employs the lysine acetylases p300/CBP and tumor suppressor Rb to repress select host genes and promote productive virus infection. Cell Host Microbe. 2014;16(5):663–76. 10.1016/j.chom.2014.10.004 . 25525796 PMC4418520

[18] Ferrari R, Pellegrini M, Horwitz GA, Xie W, Berk AJ, Kurdistani SK. Epigenetic reprogramming by adenovirus e1a. Science. 2008;321(5892):1086–8. Epub 2008/08/23. 321/5892/1086 [pii] 10.1126/science.1155546 18719284 PMC2693122

[19] Ferrari R, Su T, Li B, Bonora G, Oberai A, Chan Y, et al Reorganization of the host epigenome by a viral oncogene. Genome Res. 2012;22(7):1212–21. Epub 2012/04/14. 10.1101/gr.132308.111 22499665 PMC3396363

[20] Horwitz GA, Zhang K, McBrian MA, Grunstein M, Kurdistani SK, Berk AJ. Adenovirus small e1a alters global patterns of histone modification. Science. 2008;321(5892):1084–5. Epub 2008/08/23. 321/5892/1084 [pii] 10.1126/science.1155544 18719283 PMC2756290

[21] Bellett AJ, Li P, David ET, Mackey EJ, Braithwaite AW, Cutt JR. Control functions of adenovirus transformation region E1A gene products in rat and human cells. Mol Cell Biol. 1985;5(8):1933–9. 3837852 10.1128/mcb.5.8.1933-1939.1985 PMC366910

[22] Pelka P, Ablack JN, Shuen M, Yousef AF, Rasti M, Grand RJ, et al Identification of a second independent binding site for the pCAF acetyltransferase in adenovirus E1A. Virology. 2009;391(1):90–8. Epub 2009/06/23. S0042-6822(09)00332-8 [pii] 10.1016/j.virol.2009.05.024 . 19541337

[23] Harlow E, Franza BR Jr., Schley C. Monoclonal antibodies specific for adenovirus early region 1A proteins: extensive heterogeneity in early region 1A products. J Virol. 1985;55(3):533–46. . 3894685 10.1128/jvi.55.3.533-546.1985 PMC255001

[24] Reich NC, Sarnow P, Duprey E, Levine AJ. Monoclonal antibodies which recognize native and denatured forms of the adenovirus DNA-binding protein. Virology. 1983;128(2):480–4. . 6310869 10.1016/0042-6822(83)90274-x

[25] Pfaffl MW. A new mathematical model for relative quantification in real-time RT-PCR. Nucleic Acids Res. 2001;29(9):e45 11328886 10.1093/nar/29.9.e45 PMC55695

[26] Radko S, Koleva M, James KM, Jung R, Mymryk JS, Pelka P. Adenovirus E1A targets the DREF nuclear factor to regulate virus gene expression, DNA replication, and growth. J Virol. 2014;88(22):13469–81. 10.1128/JVI.02538-14 25210186 PMC4249066

[27] Jones N, Shenk T. Isolation of adenovirus type 5 host range deletion mutants defective for transformation of rat embryo cells. Cell. 1979;17(3):683–9. . 476833 10.1016/0092-8674(79)90275-7

[28] Haley KP, Overhauser J, Babiss LE, Ginsberg HS, Jones NC. Transformation properties of type 5 adenovirus mutants that differentially express the E1A gene products. Proc Natl Acad Sci U S A. 1984;81(18):5734–8. 6091106 10.1073/pnas.81.18.5734 PMC391785

[29] Palombella VJ, Rando OJ, Goldberg AL, Maniatis T. The ubiquitin-proteasome pathway is required for processing the NF-kappa B1 precursor protein and the activation of NF-kappa B. Cell. 1994;78(5):773–85. . 8087845 10.1016/s0092-8674(94)90482-0

[30] Spindler KR, Eng CY, Berk AJ. An adenovirus early region 1A protein is required for maximal viral DNA replication in growth-arrested human cells. J Virol. 1985;53(3):742–50. 3973965 10.1128/jvi.53.3.742-750.1985 PMC254702

[31] Miller DL, Myers CL, Rickards B, Coller HA, Flint SJ. Adenovirus type 5 exerts genome-wide control over cellular programs governing proliferation, quiescence, and survival. Genome Biol. 2007;8(4):R58 10.1186/gb-2007-8-4-r58 17430596 PMC1896011

[32] Conboy CM, Spyrou C, Thorne NP, Wade EJ, Barbosa-Morais NL, Wilson MD, et al Cell cycle genes are the evolutionarily conserved targets of the E2F4 transcription factor. PloS one. 2007;2(10):e1061 10.1371/journal.pone.0001061 17957245 PMC2020443

[33] Ren B, Cam H, Takahashi Y, Volkert T, Terragni J, Young RA, et al E2F integrates cell cycle progression with DNA repair, replication, and G(2)/M checkpoints. Genes Dev. 2002;16(2):245–56. 10.1101/gad.949802 11799067 PMC155321

[34] Lee BH, Mathews MB. Transcriptional coactivator cAMP response element binding protein mediates induction of the human proliferating cell nuclear antigen promoter by the adenovirus E1A oncoprotein. Proc Natl Acad Sci U S A. 1997;94(9):4481–6. 9114015 10.1073/pnas.94.9.4481 PMC20748

[35] Tiainen M, Spitkovsky D, Jansen-Durr P, Sacchi A, Crescenzi M. Expression of E1A in terminally differentiated muscle cells reactivates the cell cycle and suppresses tissue-specific genes by separable mechanisms. Mol Cell Biol. 1996;16(10):5302–12. 8816442 10.1128/mcb.16.10.5302 PMC231529

[36] Mymryk JS, Bayley ST. Multiple pathways for gene activation in rodent cells by the smaller adenovirus 5 E1A protein and their relevance to growth and transformation. J Gen Virol. 1993;74 (Pt 10):2131–41. Epub 1993/10/01. . 8409938 10.1099/0022-1317-74-10-2131

[37] Montell C, Fisher EF, Caruthers MH, Berk AJ. Resolving the functions of overlapping viral genes by site-specific mutagenesis at a mRNA splice site. Nature. 1982;295(5848):380–4. . 7057903 10.1038/295380a0

[38] Winberg G, Shenk T. Dissection of overlapping functions within the adenovirus type 5 E1A gene. EMBO J. 1984;3(8):1907–12. 6479152 10.1002/j.1460-2075.1984.tb02066.x PMC557616

[39] Nichols WW, Murphy DG, Cristofalo VJ, Toji LH, Greene AE, Dwight SA. Characterization of a new human diploid cell strain, IMR-90. Science. 1977;196(4285):60–3. . 841339 10.1126/science.841339

[40] Hayflick L, Plotkin SA, Norton TW, Koprowski H. Preparation of poliovirus vaccines in a human fetal diploid cell strain. Am J Hyg. 1962;75:240–58. . 13905660 10.1093/oxfordjournals.aje.a120247

[41] Malladi A, Quinlan MP. Mutations in CR1 of E1A 12S yield dominant negative suppressors of immortalization and the lytic cycle. Virology. 1997;233(1):51–62. 10.1006/viro.1997.8605 . 9201216

[42] Chatterjee PK, Vayda ME, Flint SJ. Adenoviral protein VII packages intracellular viral DNA throughout the early phase of infection. EMBO J. 1986;5(7):1633–44. 3743550 10.1002/j.1460-2075.1986.tb04406.x PMC1166989

[43] Ou HD, Kwiatkowski W, Deerinck TJ, Noske A, Blain KY, Land HS, et al A structural basis for the assembly and functions of a viral polymer that inactivates multiple tumor suppressors. Cell. 2012;151(2):304–19. 10.1016/j.cell.2012.08.035 23063122 PMC3681303

[44] Soria C, Estermann FE, Espantman KC, O'Shea CC. Heterochromatin silencing of p53 target genes by a small viral protein. Nature. 2010;466(7310):1076–81. Epub 2010/08/27. 10.1038/nature09307 20740008 PMC2929938

[45] Mailand N, Gibbs-Seymour I, Bekker-Jensen S. Regulation of PCNA-protein interactions for genome stability. Nat Rev Mol Cell Biol. 2013;14(5):269–82. 10.1038/nrm3562 . 23594953

[46] Manthei KA, Keck JL. The BLM dissolvasome in DNA replication and repair. Cell Mol Life Sci. 2013;70(21):4067–84. 10.1007/s00018-013-1325-1 23543275 PMC3731382

[47] Nimonkar AV, Genschel J, Kinoshita E, Polaczek P, Campbell JL, Wyman C, et al BLM-DNA2-RPA-MRN and EXO1-BLM-RPA-MRN constitute two DNA end resection machineries for human DNA break repair. Genes Dev. 2011;25(4):350–62. 10.1101/gad.2003811 21325134 PMC3042158

[48] Obert S, O'Connor RJ, Schmid S, Hearing P. The adenovirus E4-6/7 protein transactivates the E2 promoter by inducing dimerization of a heteromeric E2F complex. Mol Cell Biol. 1994;14(2):1333–46. Epub 1994/02/01. 8289811 10.1128/mcb.14.2.1333-1346.1994 PMC358488

[49] Schaley JE, Polonskaia M, Hearing P. The adenovirus E4-6/7 protein directs nuclear localization of E2F-4 via an arginine-rich motif. J Virol. 2005;79(4):2301–8. Epub 2005/02/01. 79/4/2301 [pii] PubMed Central PMCID: PMC546583. 15681431 10.1128/JVI.79.4.2301-2308.2005 PMC546583

